# Biomaterials Loaded with Growth Factors/Cytokines and Stem Cells for Cardiac Tissue Regeneration

**DOI:** 10.3390/ijms21175952

**Published:** 2020-08-19

**Authors:** Saltanat Smagul, Yevgeniy Kim, Aiganym Smagulova, Kamila Raziyeva, Ayan Nurkesh, Arman Saparov

**Affiliations:** Department of Medicine, School of Medicine, Nazarbayev University, Nur-Sultan 010000, Kazakhstan; ssmagul@nu.edu.kz (S.S.); Yevgeniy.Kim@nu.edu.kz (Y.K.); Aiganym.Smagulova@nu.edu.kz (A.S.); kamila.raziyeva@nu.edu.kz (K.R.); ayan.nurkesh@nu.edu.kz (A.N.)

**Keywords:** biomaterials, stem cells, cytokines, growth factors, cardiac tissue regeneration, regenerative medicine

## Abstract

Myocardial infarction causes cardiac tissue damage and the release of damage-associated molecular patterns leads to activation of the immune system, production of inflammatory mediators, and migration of various cells to the site of infarction. This complex response further aggravates tissue damage by generating oxidative stress, but it eventually heals the infarction site with the formation of fibrotic tissue and left ventricle remodeling. However, the limited self-renewal capability of cardiomyocytes cannot support sufficient cardiac tissue regeneration after extensive myocardial injury, thus, leading to an irreversible decline in heart function. Approaches to improve cardiac tissue regeneration include transplantation of stem cells and delivery of inflammation modulatory and wound healing factors. Nevertheless, the harsh environment at the site of infarction, which consists of, but is not limited to, oxidative stress, hypoxia, and deficiency of nutrients, is detrimental to stem cell survival and the bioactivity of the delivered factors. The use of biomaterials represents a unique and innovative approach for protecting the loaded factors from degradation, decreasing side effects by reducing the used dosage, and increasing the retention and survival rate of the loaded cells. Biomaterials with loaded stem cells and immunomodulating and tissue-regenerating factors can be used to ameliorate inflammation, improve angiogenesis, reduce fibrosis, and generate functional cardiac tissue. In this review, we discuss recent findings in the utilization of biomaterials to enhance cytokine/growth factor and stem cell therapy for cardiac tissue regeneration in small animals with myocardial infarction.

## 1. Introduction

Cardiovascular diseases (CVD) are the leading cause of mortality worldwide [1,2]. In 2017, about 17.8 million deaths globally were attributed to CVD and in the U.S. alone, CVD, which include heart disease and stroke, were among the top ten causes of death, accounting for 74% of total deaths [3]. Coronary heart disease causes the majority of deaths in CVD, with myocardial infarction (MI) often leading to heart failure. Tissue damage at the site of infarction triggers local inflammation that attracts neutrophils and monocytes to clear the area of cell debris and produce reactive oxygen species. Migration of monocytes with reparative functions induces the formation of new vasculature and collagen production and eventually, leads to tissue repair and fibrotic tissue formation [4,5,6]. One biomedical approach for improving cardiac tissue regeneration is the delivery of therapeutic growth factors and cytokines [7]. Growth factors and cytokines have attracted the attention of researchers and clinicians due to their angiogenic and antiapoptotic properties, as well as their ability to increase cell proliferation and mobilize endogenous cell migration [8]. Various factors and cytokines, including but not limited to, tumor necrosis factor-α (TNF-α) and interleukin-8 (IL-8), are also upregulated in MI and participate in triggering inflammatory cascade. Therefore, regulation of pro- and anti-inflammatory mediator functions can be used to ameliorate inflammation and to facilitate cardiac tissue regeneration [9]. However, there are some challenges associated with growth factors/cytokines. For example, the systemic administration of growth factors/cytokines is not efficient due to a short in vivo half-life and poor bioavailability at the target sites. This, in turn, requires repeated injections, resulting in more side effects and greater treatment costs [10,11]. Moreover, simultaneous and rapid diffusion can lead to formation of immature and unstable blood vessels in the case of therapy with angiogenic growth factors [12].

Biomaterials offer a controlled and sustained release of bound growth factors and cytokines, which makes them a promising tool for overcoming the aforementioned challenges [13,14]. Biomaterials of natural, synthetic or hybrid origins were developed. They demonstrated therapeutic benefits when used either alone or when loaded with agents such as growth factors, cytokines or stem cells [15]. The use of biomaterials alone exerts positive effects on cardiac tissue regeneration, possibly via mimicking the extracellular matrix (ECM) and providing direct mechanical support. Some biomaterials also help to increase electrical conductance in a fibrotic scar region, which is important for normal functioning of the heart [16,17].

The endogenous regenerative capacity of cardiac tissue is limited: adult cardiomyocyte proliferation, cardiac stem cell activation, and bone marrow progenitor cell migration are not efficient enough to regenerate fully functional cardiac tissue. Post-MI repair often involves tissue replacement with non-functional fibrotic scarring, which can later lead to heart failure. For these reasons, stem cell therapy is considered a promising approach in MI treatment, being particularly beneficial for reducing the infarcted area and promoting cardiac function recovery [18]. Different stem cell sources such as mesenchymal stem cells (MSCs), cardiac stem cells (CSCs), induced pluripotent stem cells (iPSCs), and others are now recognized for their potential use in cardiac tissue regeneration [19]. Stem cell benefits in MI treatment include differentiation capacity, stimulation of resident CSCs, reduction in inflammation, and ability to provide structural support by connective tissue formation and fibroblast differentiation [20]. Release of cytokines and growth factors by stem cells allows for immunomodulation, angiogenesis, and stimulation of adjacent cells via paracrine mechanisms [21,22]. However, harsh conditions at the infarction site present a significant burden for stem cell survival. These conditions include, but are not limited to, hypoxia, fibrogenesis, low blood supply, and inflammation [23]. Therefore, biomaterials can serve as a stem cell delivery system that increases the living potency of the cells after transplantation and enhance the exerted effects. This review will focus on recent findings on the use of biomaterials as drug delivery systems for growth factors, cytokines, and stem cells for improving cardiac tissue regeneration in small animal models of MI.

## 2. Biomaterials Loaded with Growth Factors and Cytokines for Cardiac Tissue Regeneration

The use of biomaterials is now rapidly evolving as a new approach for MI treatment [24,25]. They are composed of a plethora of various polymers and can be used as a drug delivery system in the field of regenerative medicine [26]. The most common types are polymeric micro and nanospheres, nanoparticles (NPs), nanofibrous structures, coacervates, hydrogels, cryogels, and scaffolds. They differ in their size and assembling materials, as well as in their morphology, i.e., sheet versus vesicle-like structures [27,28,29,30,31,32,33]. Hydrogels, in particular, are widely investigated in the area of CVD. Hydrogel is largely composed of water and a cross-linked polymer and physically resembles tissue [34]. Hydrogels made of cardiac ECM, alginate, hyaluronic acid (HA), natural biomaterials (collagen, fibrin, and heparin), synthetic polymers, and microparticles have been studied pre-clinically for cardiac repair [35]. The effects of hydrogel administration include direct mechanical strengthening [36], enhanced angiogenesis and regeneration of myocardial tissue, reduced apoptosis and scar size, and improved cardiac function recovery [37]. Moreover, multiple studies showed that the use of biomaterials alone favorably affects various cells in the post-MI environment such as macrophages, cardiomyocytes, fibroblasts, and endothelial cells [38]. Recently, hydrogels made of ECM-based biomaterials have drawn attention because of their ability to mimic native ECM and minimize immunogenicity [39]. McLaughlin and colleagues treated mice at the end of the proliferative phase of wound healing with the injectable biomaterial, which contained human recombinant collagen I and III, one of the main proteins in the ECM of heart tissue. The treatment reduced inflammation, polarized macrophages towards M2 phenotype, increased capillary density at the border zone, and improved cardiac function [40]. The application of the self-assembling peptide (SAP) cell-free hydrogel also significantly improved the functionality of the heart post-MI through increased angiogenesis and reduced scar formation [41]. The beneficial effects of biomaterials are shown to depend on the time of therapy administration. In the study by Blackburn and colleagues, 3h post-MI application of collagen-based hydrogel in a murine model reduced cell apoptosis as well as increased capillary density and as a result, improved left ventricular ejection fraction. The authors also reported that biomaterial therapy is ineffective after 14 days post-MI [37]. The mechanisms of the exerted effects of biomaterials are possibly mediated by modifying the inflammatory immune response. It was demonstrated that hydrogel treatment also reduced the number of macrophages and TNF-α production in cardiac tissue. The in vitro culture of macrophages on biomaterials demonstrated a decrease in pro-inflammatory cytokines and an increase in anti-inflammatory cytokines [37].

Fibrosis, and its consequent non-functional scar formation, is considered to be a major problem following MI, leading to left ventricle remodeling and heart failure. Several biomaterials were designed to improve conduction of electrical signals in the scar region. For example, pyrrole was grafted onto a chitosan biomaterial to produce a conductive polypyrrole (PPy)-chitosan hydrogel. In vivo experiments used a coronary artery ligation rat model of acute MI to show reduced QRS complex on an electrocardiogram and improved transverse conduction velocity in PPy-chitosan group. It was demonstrated that both chitosan alone and PPy-chitosan were effective in preserving heart function, but PPy-chitosan further improved the indices, suggesting better maintenance of heart function as compared to a non-conductive biomaterial [16]. Cui and colleagues tested PPy-chitosan in a cryoablation injury rat model and reported a significant improvement in longitudinal conduction velocity in comparison to the chitosan only group. Electromyography was used to assess the conductivity of scar tissue ex vivo, which showed a significant 300–350% increase in electrical signals in the myocardial scar tissue in the group treated with PPy-chitosan [42].

Extensive research has been performed to study the importance of growth factors, cytokines, and different components of ECM in the treatment of MI [43,44]. It was shown that transforming growth factor-β (TGF-β) stimulates both Smad3-dependent and independent activation of macrophages, with the involvement of Smad3 in phagocytosis activation, secretion of vascular endothelial growth factor (VEGF) and TGF-β1, and protection against adverse cardiac tissue remodeling [45]. IL-10 is also important because its deficiency increases necrosis and neutrophil migration, with an enlargement in infarct size. Moreover, IL-10 deficiency impairs the ability of endothelial progenitor cells to suppress cell apoptosis, reduce scar size, increase neovascularization, and improve left ventricle remodeling, which is mediated by upregulation of integrin-linked kinase [46]. In contrast, treatment with IL-10 suppresses inflammation, polarizes macrophages towards M2 phenotype, activates fibroblasts, and improves left ventricle remodeling [47]. Another important growth factor is VEGF, which can be released from cardiac macrophages to simulate angiogenesis and heart muscle repair by regulating endothelial cell proliferation, migration, and apoptosis [43,48]. Furthermore, VEGF-A, fibroblast growth factor (FGF), and stromal cell-derived factor-1 (SDF-1) can stimulate neovascularization [49]. IL-4 is also a key cytokine because IL-4 administration differentiates macrophages, which are derived from Ly6C^high^ monocytes, into a M2 phenotype [50]. However, application of growth factors and cytokines in clinical practice is hindered by their short half-lives, decreased stability, and deactivation by enzymes [27]. For example, the half-life of VEGF is approximately thirty-four minutes in plasma [51]. Therefore, biomaterials can serve as promising tools for the protection, delivery, and sustained release of growth factors and cytokines [52]. Table 1 summarizes the use of biomaterials loaded with growth factors and cytokines for cardiac tissue regeneration.

**Figure 1:**
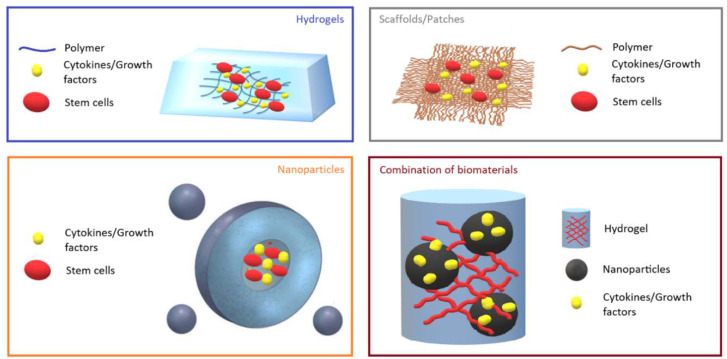
Representative images of biomaterials. Hydrogels, scaffolds/patches, and nanoparticles loaded with growth factors/cytokines and stem cells, and their combination are shown.

The incorporation of growth factors and cytokines into engineered biomaterials, such as hydrogels and NPs, offers even more opportunities for MI therapy (Figure 1). As an example, the injection of heparan sulfate proteoglycans (HSPG), which is a major component of ECM, with basic FGF (bFGF), extended the bioavailability of the growth factor by protecting it from degradation, and improved angiogenesis and cardiac function in animals with MI [53]. Another group also used bFGF that was fused with glutathione-S-transferase (GST) and matrix metalloproteinase (MMP)-2/9 cleavable peptide TIMP, and then, incorporated the complex into a glutathione-modified collagen hydrogel. This approach allowed for the controlled release of bFGF after TIMP was cleaved by the secreted MMP-2/9 at the site of tissue infarction. The use of this type of hydrogel decreased collagen deposition, increased vascularization, and improved heart function in rats with MI [54]. The mechanism of bFGF, which is a paracrine signaling protein, is mediated through binding to FGF receptor-heparan sulfate complex and further activation of tyrosine kinase. Downstream signaling proceeds via RAS-mitogen-activated protein kinase RAS-(MAPK) and phosphatidylinositide 3-kinase (PI3K) pathways [55]. In a separate study, sustained and targeted delivery of neuregulin-1β (NRG), which is a member of epidermal growth factor that regulates cardiomyocyte development and proliferation, by a hydroxyethyl methacrylate hyaluronic acid (HEMA-HA) hydrogel, demonstrated a cardioprotective effect and significantly improved ventricular function and structure [38]. The cardioprotective effect was assessed by the amount of caspase-3 in murine hearts post-MI, which was significantly reduced in the NRG-hydrogel group in comparison to the control groups treated with phosphate-buffered saline, NRG, or hydrogel alone. Caspase-3 is a key mediator of the terminal apoptotic pathway and its downregulation is associated with reduced infarct size, decreased apoptotic index of myocytes, and enhanced heart function in an experimental model of MI [56]. Awada and colleagues demonstrated that sequential delivery of VEGF followed by platelet-derived growth factor (PDGF) using a fibrin gel/heparin coacervate delivery system improves angiogenesis and cardiac function and reduces scar formation and inflammation at two and four weeks after MI in a rat model [57]. Mechanistically, VEGF promotes angiogenesis by activating or affecting different pathways and proteins, including PI3K, VRAP, Src tyrosine kinase, MAPK, and phospholipase C [58]. Recent reports show the critical role of multiple types of tyrosine and serine/threonine phosphatases, such as Shp2 and low molecular weight protein tyrosine phosphatase, in negative/positive regulation of VEGFR-2 signaling [59]. Interestingly, although VEGF demonstrated positive effects on MI in the experimental animal models, the results were not very promising according to several clinical trials [49]. One possible reason is the short period of protein bioactivity in vivo [60].

Although natural hydrogels are widely used in experiments [61], synthetic and hybrid hydrogels are also broadly investigated [62]. Synthetic glycosaminoglycan mimetic peptide nanofiber developed by Rufaihah and colleagues promoted the formation of new blood vessels and the differentiation of cardiomyocytes in rats [63]. Carlini and colleagues designed synthetic cyclic SAPs that were delivered to the heart through a catheter and rapidly formed a hydrogel after cleavage by enzymes MMP-2/9 and elastase, which are endogenous to the site of infarction in a rat model of MI. In addition to their low viscosity and ability to form a gel-like structure, the novel SAPs showed hemocompatibility, biocompatibility, and non-thrombogenicity that open up the possibility for implementation in drug delivery for the treatment of MI [35]. A novel hybrid temperature-responsive poly(N-isopropylacrylamide) gelatin-based injectable hydrogel was developed for cardiac tissue engineering and it exhibited a high level of cardiomyocyte and cardiac fibroblast survival and enhanced cytoskeletal organization [64]. Moreover, myeloid-derived growth factor (Mydgf) was incorporated into an injectable citrate-based polyester hydrogel to investigate its effects on improving cardiac tissue repair following MI. The combination of the released Mydgf and citrate, which is an important substrate in cellular energy metabolism, reduced cell apoptosis and scar formation as well as improved angiogenesis and cardiac function [65]. In the study by Waters and colleagues, therapeutic biomolecules, such as growth factors and cytokines, secreted by human adipose-derived stem cells (ADSCs), were loaded into laponite/gelatin hydrogel and injected into the peri-infarct region in an acute MI rat model, which resulted in increased angiogenesis and reduced fibrosis as well as a significant improvement in ejection fraction and cardiac output [66]. The hydrogel could accommodate growth factors due to laponite, which is a synthetic nanoclay composed of discoid NPs that can bind growth factors through electrostatic forces.

Along with hydrogels, nanoscale carriers (Table 1) are extensively studied for cardiac tissue repair following MI [67]. Targeted delivery, maintenance of protein stability, presence in blood circulation for an extended time, and controlled release of loaded agents make NPs attractive carriers for cardiac tissue therapy. For the purpose of targeting MI, Nguyen and colleagues developed NPs that respond to a specific enzymatic stimulus of MMP-9 and MMP-2 enzymes, which are upregulated upon infarction. This method allows for better accumulation at the MI site and longer clearance from the system [68]. Moreover, DNA enzymes conjugated to gold NPs have been demonstrated to produce an anti-inflammatory effect and improve cardiac function in a rat model of acute MI via silencing TNF-α and downregulating pro-inflammatory mediators, such as IL-12β, IL-1β, IL-6, as well as inducible nitric oxide synthase [69]. Another group loaded liraglutide in poly(lactic-co-glycolic acid)-poly(ethylene glycol) nanoparticles (NP-liraglutide) and delivered it to the infarcted rats via intramyocardial injection to overcome the challenges posed by its short half-life [70]. As a result, the NP-liraglutide system is retained in the myocardium over four weeks, thus, enhancing heart function, attenuating adverse cardiac remodeling, stimulating angiogenesis, and suppressing cardiomyocyte apoptosis. Although NPs appear to be a promising drug delivery system, the main concerns are their toxicity and tendency to aggregate, which lead to changes in physical and chemical properties and the formation of protein corona on the surface of NPs that prevents specific targeting [67].

Hydrogels and NPs can be used separately, as previously mentioned, or in combination. For example, a sulfonated hydrogel incorporated with VEGF and IL-10 and combined with PDGF-loaded micelle NPs showed a sequential and sustained release of all three factors for 28 days in vitro and a significant increase in the formation of mature vessels in vivo on a subcutaneous injection murine model [12]. As a result, this novel system significantly promoted angiogenesis and demonstrated the potential to ameliorate inflammation for improving cardiac repair post-MI. Another study used a novel, shear-thinning biocompatible and catheter-deliverable HA-based hydrogel loaded with dimeric fragment of hepatocyte growth factor (HGFdf) and a variant of stromal cell-derived factor 1α (ESA) to demonstrate a dual stage release that decreased infarct size and improved angiogenesis and heart function following MI [71]. ESA is a potent chemokine that attracts endothelial progenitor cells to infarcted areas and displays significant pro-angiogenic and wound healing effects. Moreover, hepatocyte growth factor prevents tissue fibrosis by inhibiting TGF-β production and stimulating MMP-1 to increase collagen degradation, as well as possessing pro-angiogenic and cardiomyogenic properties [72].

Another type of biomaterial is a cardiac patch that is directly applied to the myocardium. An acellular epicardial patch, developed from hydrogel, was also shown to prevent left ventricle remodeling and improve cardiac function in acute and subacute MI models in rats [73]. Wan and colleagues developed a novel cardiac patch derived from human heart valves. It is thought that the use of a human heart valve-derived scaffold (hHVS) may be superior to other approaches in cardiac repair by providing a native myocardial ECM. An in vitro study showed increased cellular proliferation and induction of cardiomyogenic differentiation of cells attached to a hHVS. An in vivo experiment demonstrated that patch application of hHVS alone reduced infarct size in a murine MI model. However, c-kit+ stem cell-seeded hHVS was more effective [74]. Cardiac patches have also been used for growth factor delivery (Table 1). Rodness and colleagues demonstrated that VEGF-containing calcium-alginate microsphere patches increased capillary density and improved tissue regeneration and cardiac function [75]. Transplanted human cardiomyocyte patches, which contained cardiomyocytes derived from human iPSCs and NPs loaded with FGF1 and CHIR99021, an inhibitor of the enzyme glycogen synthase kinase-3, reduced infarction size and improved angiogenesis and cardiac function. The combination of these factors reduced apoptosis and increased proliferation of transplanted cardiomyocytes [76].

In summary, biomaterials including micro and nanospheres, lipid NPs, nanofibrous structures, coacervate, hydrogels, and scaffolds appear to be a promising drug delivery system for cardiac tissue repair following MI. They can be administered alone or loaded with powerful therapeutic agents, such as growth factors and cytokines, that regulate cardiac tissue regeneration following MI. Biomaterials loaded with growth factors/cytokines have been shown to enhance angiogenesis and tissue regeneration, reduce cardiac cell death and scar size, ameliorate inflammation, and improve cardiac function (Table 1).

## 3. Biomaterials Loaded with Stem Cells for Cardiac Tissue Regeneration

Stem cells possess self-regenerating, differentiating, and immunomodulating properties, as well as release trophic factors. Therefore, they have been considered to be promising tools for cardiac tissue regeneration [20,77,78]. Many reports have demonstrated the therapeutic potential of various stem cell types, such as bone marrow-derived stem cells (BMSCs), ADSCs, cardiac-derived stem cells/cardiac progenitor cells (CPCs), and others, on myocardial tissue regeneration [79,80,81,82,83]. Moreover, stem cells have shown their therapeutic efficiency in several clinical trials [84]. Treatment with MSCs can improve left ventricle remodeling and function through decreasing scar size, promoting angiogenesis, and improving contractility [85,86]. Stem cells mediate cardioprotection by lowering the number of apoptotic myocytes at the site of injection. The mechanism responsible for protection includes insulin-like growth factor 1 (IGF-1)-mediated activation of stress-signaling and inflammatory response pathways and the suppression of cardiac transcription factor, nuclear factor kappa B [20]. Stem cells also support neoangiogenesis in post-MI tissue through positive regulation of VEGF, angiopoietin-1 (Ang-1), epidermal growth factor (EGF), and PDGF. Cell survival and proliferation is regulated by the AKT signaling pathway [87]. Despite the beneficial effects of stem cells on post-MI tissue regeneration, limitations such as low engraftment and survival rates in a harsh microenvironment compromise the clinical translatability of this approach [88]. Poor engraftment of transplanted cells is linked to mechanical loss during injection, loss of viability during long-lasting pre-conditioning, hypoxia, nutritional deficiencies, and low cell proliferation rate in vivo [88]. Therefore, various approaches are now being examined to increase engraftment and enhance the survival and stability of stem cells. One such approach is the use of biomaterials. Table 2 summarizes the use of biomaterials loaded with stem cells for cardiac tissue regeneration.

Several stem cell delivery systems are now utilized, including direct needle injection, nanogels, polymers, and inorganic nanomaterials [89,90,91]. Needle injection is the preferred method in clinics as it is less invasive. However, it has low cell retention, with less than 5% of transplanted cells reaching and remaining in cardiac tissue [92]. Recently, Park and colleagues proposed a new efficient direct MSC injection method to treat MI. MSCs were used in favor of other stem cell types based on their efficiency in reducing apoptosis and inflammation, as well as their ability to enhance vascularization and cardioprotection. In their study, they applied electrostatic interactions between bioengineered cationic mussel adhesive protein (MAP) and anionic HA. The resulting MAP/HA coacervate, named the adhesive protein-based immiscible condensed liquid system (APICLS), was successfully loaded with MSCs. APICLS was shown to be an innovative platform to treat MI, where stem cells demonstrated higher viability and retention and therefore, recovered infarcted tissue more effectively [92]. Another promising biomaterial for in vivo stem cell delivery is a collagen-based hydrogel transglutaminase cross-linked gelatin (Col-Tgel). The Col-Tgel-ADSCs system was shown to greatly improve MI treatment by enhancing engraftment of stem cells. ADSCs, which are the MSCs derived from adipose tissue, are shown to have several advantages over the bone marrow-derived MSCs. These include a more attractive cost and yield, a less invasive method for isolation, and a higher rate of cell growth [93]. In the study by Blocki and colleagues, injectable microcapsules made of agarose and ECM components were developed to enhance the survival of bone marrow-derived MSCs after their transplantation to rats with acute MI. The design was safe and efficient as evidenced by the absence of fibrotic response and persistence of the cells in the infarcted myocardium for four weeks after injection. In contrast, when these cells were injected without microcapsules, i.e., as cell suspension, they were detectable in post-MI hearts for only two days following transplantation [94]. Gallagher and colleagues showed that delivering MSCs using an arginylglycylaspartic acid (RGD)-modified HA hydrogel improves MSCs survival in the ischemic area. This effect was achieved due to HA being a natural ECM component and RGD being a tripeptide sequence that promoted MSC attachment to the hydrogel [95]. Another study successfully improved post-MI heart recovery in rats by delivering iPSCs in erythropoietin-linked hydrogel. The hydrogel was administered by injection into the myocardium [96]. Moreover, Cai and colleagues developed a novel designer self-assembling peptide (DSAP) consisting of the existing synthetic SAP and angiopoetin-1-derived pro-survival peptide QHREDGS in order to improve engraftment and retention of MSCs. This system significantly improved the survival of rat MSCs when they were injected into rats with MI [97]. Enhanced cell survival could be attributed to the presence of the QHREDGS peptide in the SAP. This peptide is an integrin-binding motif of Ang-1, a growth factor that stimulates endothelial cell survival, migration, and differentiation [98]. It was shown that QHREDGS peptide could mediate the same effects on its own, without being a part of Ang-1, when it is incorporated into various biomaterials such as hydrogels, for example [97]. However, the exact mechanism by which it promotes cell survival is still to be elucidated.

Pro-survival peptides were also used in the study by Lee and colleagues. In particular, they utilized collagen–dendrimer biomaterial crosslinked with pro-survival peptide analogues, namely, bone morphogenetic protein-2 peptide analogue, erythropoietin peptide analogue, and FGF2 peptide analogue, to augment the survival of CPCs in the MI model of mice [99]. CPCs that were transplanted with pro-survival factors enriched the collagen matrix and showed significantly greater long-term survival and engraftment compared to cells without the matrix. The authors described the molecular mechanism of enhanced cellular survival. Thus, the pro-survival matrix caused an increase in the expression of genes involved in the MAPK and phosphatidylinositol-3-OH kinase-protein kinase B (PI3K-AKT) pathways, while inhibiting pro-apoptotic pathways. In another study, silica-coated magnetic nanoparticles (MNPs) and an external magnet were utilized to enhance the survival of transplanted cells [100]. Embryonic cardiomyocytes, embryonic stem cell-derived cardiomyocytes, and BMSCs were incorporated into MNPs. Afterwards, the cell-MNP delivery system was intramyocardially injected into a murine model of MI, and a magnet was placed close to the chest of the animals to force the cells into the infarcted tissue. The treatment had drastically enhanced cell engraftment by 7-fold and 3.4-fold, two and eight weeks after application, respectively. The increased engraftment of the transplanted cells was due to a decrease in the loss of cells via the injection channel, which increased their proliferation and reduced apoptosis. The graphene oxide/alginate microgels constructed for cell delivery also demonstrated a favorable approach to promote MI recovery of the left ventricle during transplantation of MSCs [101].

Cardiac cell patches (Table 2) can be constructed from natural or synthetic materials, albeit natural materials are more favorable due to their biocompatibility and comparatively low cost [102]. Studies show that cardiac patches loaded with stem cells, where MSCs are preferable compared to CPCs, embryonic or iPSCs, facilitate a higher engraftment rate of transplanted cells. Moreover, cell patches also provide a positive impact on cardiomyogenesis and angiogenesis [102,103]. Wang and colleagues transplanted poly(ε-caprolactone)/gelatin patch loaded with MSCs into the epicardium of the murine model of MI. The patch reduced MI-induced damage by promoting angiogenesis, lymphangiogenesis, and cardiomyogenesis, decreasing scar size and enhancing the release of paracrine factors from stem cells. They also showed an increase in the expression of hypoxia-inducible factor 1α, TGF-β, VEGF, and SDF1 factors and a negative regulation of CXCL14. Cytokine release enhanced the recruitment of endogenous c-kit+ cells and activated the epicardium [103]. Chen and colleagues designed a novel chitosan and silk fibroin microfibrous cardiac patch that significantly improved the survival of murine adipose tissue-derived MSCs in infarcted hearts of a rat model [104]. This was achieved due to the structural resemblance of the patch to the native ECM of the heart. Thus, the patch provided a suitable environment for the retention and survival of the transplanted cells. Nevertheless, the detailed mechanism of this process is yet to be identified. Similarly, in the study by Gaetani and colleagues, it was shown that a 3D-printed HA/gelatin cardiac patch could support long-term survival and differentiation of the CPCs when they were tested on the mouse model of MI [105]. Su and colleagues used a cardiac patch not only to provide an adhesion and retention framework for stem cells, but to also nutritionally support them [106]. Specifically, they developed a vascularized fibrin gel that could accommodate CSCs. Such a construct would help stem cells receive nutrients through biomimetic blood vessels (BMV) within the hydrogel and consequently, enrich their survival. In addition, the BMV were made of fibronectin, a constituent of the natural ECM, and hence, provided the appropriate environment for the transplanted cells. Moreover, Dong and colleagues constructed a patch made of gold NPs coated with a combination of ECM and silk proteins [107]. The patch was loaded with rat bone marrow-derived MSCs and tested in a cryoinjury model of MI in rats. The construct was found to greatly improve stem cell survival and retention as well as significantly decrease the infarct size 28 days post-infarction. The authors proposed several mechanisms to achieve beneficial effects of the patch on cell viability. Namely, the construct possesses antioxidant properties and acts as a mechanical scaffold, thus, protecting the transplanted cells from the harsh environment in the infarcted region. Gao and colleagues used an ECM scaffold to deliver human iPSC-derived cardiomyocytes, smooth muscle cells, and endothelial cells to mice with MI [108]. This treatment significantly reduced infarct size and improved cell proliferation, cardiac function, and angiogenesis. Furthermore, Tang and colleagues developed new microneedle patches loaded with cardiac stromal cells (CSCs) for post-MI tissue regeneration. Poly(vinyl alcohol)-made microneedles served as channels between myocardial tissue and regenerative factors released from CSCs. In vivo studies on a rat MI model showed that microneedle patches could promote angiogenesis, reduce fibrosis, and repair the left ventricular wall [109]. Combinatorial dual stem cell delivery is another approach to enhance the survival of transplanted stem cells. Park and colleagues used MSCs seeded on polycaprolactone patch and iPSC-derived cardiomyocytes for in vivo treatment of the rat MI model. Analysis with immunohistochemistry, gene expression, and echocardiography demonstrated significant enhancement in MI recovery. Cardiomyocytes contributed to myocardium regeneration, while growth-promoting paracrine factors from MSCs accelerated angiogenesis as well as caused iPSC-cardiomyocytes to resemble adult-like cardiomyocyte morphology [110]. Interestingly, the cardiac patch can be 3D printed for iPSC-derived cell delivery to effectively enhance post-MI treatment [111].

Another positive effect of biomaterials on stem cell therapy is the enhanced release of paracrine factors produced by the cells. Melhem and colleagues developed a microchanneled hydrogel patch that can sustain a continuous release of stem cell synthesized factors [112]. The patch was loaded with human bone marrow-derived MSCs and tested in vitro and in the murine model of MI. Patch-protected MSCs released a variety of angiogenic, anti-inflammatory, cardioprotective, antifibrotic, and antiapoptotic factors in vitro. Furthermore, the sustainable release of paracrine factors by the system was confirmed by the assessment of the VEGF release profile for one week. Over this period of time, the amount of VEGF linearly increased. The microchanneled hydrogel patch loaded with MSCs showed other benefits as well. Namely, mice treated with the patch showed significant improvement in cardiac function, which was established by echocardiographic examinations of ejection fraction and stroke volume five weeks after infarction. Importantly, the therapeutic effects of the treatment were significantly greater with MSCs, the patch without MSCs, or MSCs alone as compared to the patch without microchannels. Moreover, the effects of the patch did not depend on the number of transplanted cells, implicating that the construct could reduce the number of stem cells required for treatment. Similarly, Mayfield and colleagues showed that single cell hydrogel microencapsulation of human CSCs significantly improves the production of pro-angiogenic/cardioprotective cytokines, angiogenesis, and angiogenic cells recruitment after direct intramyocardial injection into mice with MI [113]. Less is known about biomaterial distribution after injection in vivo; Ahmadi and colleagues reported that a collagen matrix is retained mostly in the injected area with minimal distribution to non-target areas [114]. Han and colleagues utilized iron NPs that were co-cultured with rat cardiomyoblasts to boost the therapeutic efficiency of human bone marrow-derived MSCs [115]. The modified MSCs showed increased expression of various paracrine factors, namely, bFGF, HGF, VEGF, Ang-1, urokinase type plasminogen activator, placental growth factor, and monocyte chemoattractant protein-1. Moreover, pre-treated MSCs reduced infarct size, prevented fibrosis, decreased apoptosis of myocardial cells, increased angiogenesis, and improved cardiac function and the survival of rats with acute MI overall. The authors stated that improvements in the therapeutic potential of MSCs should be attributed to the increased expression of connexin 43 gap junction protein by cardiomyoblasts, which was stimulated by iron NPs. The greater expression of connexin, in turn, leads to a more efficient electrophysiologic and paracrine crosstalk between MSCs and cardiomyoblasts [116,117,118].

Yet another advantageous effect of biomaterials on stem cell treatment is their ability to accommodate factors that could act synergistically with stem cells, thus, enhancing their therapeutic actions [119,120]. For instance, Yokoyama and colleagues tested the efficiency of statins and human ADSCs combinations incorporated into NPs [121]. The treatment was injected into the tail vein of mice with MI, and its therapeutic effects were assessed for four weeks after infarction. The statin-ADSCs encapsulating NPs significantly increased the ejection fraction and several other parameters, which reflect left ventricular function. This positive effect of statin-ADSCs combination was superior compared to the use of statins or ADSCs alone. The mechanism by which the treatment brought about these improvements is likely through stimulation of the sustained and localized release of the statins by ADSCs. This, in turn, resulted in the inhibition of local inflammation, promotion of circulating stem cell recruitment, and stimulation of their differentiation to cardiomyocytes and angiogenesis [121]. Importantly, in this study, treatment efficiency was achieved with a smaller cell number of ADSCs than has ever been reported. A conductive hydrogel was also used to deliver plasmid DNA encoding endothelial nitric oxide synthase and ADSCs by injection into the infarcted myocardium. The results again demonstrated improved cardiac function with the conductive hydrogel [17]. Yao and colleagues also combined adipose-derived MSCs with a nitric oxide (NO) releasing system [122]. They utilized a naphthalene hydrogel that could maintain a controllable release of NO. In addition to demonstrating an excellent cell survival rate, the hydrogel stimulated the synthesis of angiogenic factors VEGF and SDF-1α in the MI model of mice. In yet another study, the therapeutic benefits of ADSCs were enhanced by NRG1 growth factor [123]. The ADSCs-NRG1 mixture was encapsulated into microparticles and injected into rats with MI. This combination improved cell survival as demonstrated by the persistence of the transplanted cells at three months after injection. Furthermore, ADSCs induced the shift of macrophages found in the infarcted myocardium from pro-inflammatory M1 to regenerative M2 phenotype. At the same time, NRG1 reduced the infarct size and stimulated cardiomyocyte proliferation. Compared to a separate administration, the combined treatment with ADSCs-NRG1 microparticles resulted in a more pronounced regeneration of the damaged myocardium. Chung and colleagues showed that cardiac patch-supported co-transplantation of CSCs and VEGF had a synergistic effect on angiogenesis, cell proliferation, and the recruitment of stem cells [124]. In particular, they developed a poly(l-lactic acid) mat and loaded it with rat CSCs and VEGF. When the system was tested in the rat MI models, it had greater angiogenic and cardiomyogenic effects compared to either VEGF with the patch or CSCs with the patch. Thus, numerous developments have been achieved in recent years in the usage of biomaterials to deliver stem cells to the infarction site. These include coacervates, various modifications of the hydrogels, NPs, and cardiac patches. Moreover, the combination of NPs and hydrogels also promoted transplanted cell survival. Stem cells derived from different sources were loaded into biomaterials alone, preconditioned or loaded in combination with bioactive molecules. Transplanted or tail vein injected stem cells delivering biomaterials have significantly enhanced recovery of the MI in small animal models and show promising results for their therapeutic applications. Small animals are commonly used in cardiovascular research due to their small size, low cost, short gestation time, and ease in maintenance and genetic manipulations [125]. However, there are limitations to their use that are responsible for their high failure rates in human clinical trials. These include a small heart size and anatomical differences in the coronary artery and conduction system. [126,127,128].

## 4. Conclusions

Biomaterials are being actively investigated for their use in tissue engineering and regenerative medicine due to their biodegradability and biocompatibility properties. Another important property of biomaterials is their ability to incorporate various growth factors and cytokines and to spatially and temporally control their release. Thus, biomaterials can serve as a good platform for the controlled and sustained delivery of growth factors and cytokines to ameliorate inflammation, improve angiogenesis, reduce fibrosis, and generate functional cardiac tissue. Moreover, biomaterials can be used to address some of the challenges associated with stem cell therapy of cardiovascular diseases. Specifically, they can improve stem cell survival and retention, enhance the delivery of the factors produced by the cells, support differentiation, and boost their therapeutic efficacy overall. However, despite the promising results of biomaterials in MI treatment, additional studies should be performed to improve their biocompatibility and biodegradability. Furthermore, the best source of transplanted stem cells and optimal doses of various growth factors and cytokines should be determined in order to create functional cardiac tissue and improve heart function. However, small animals do not fully recapitulate all the aspects of disease phenotypes, although they do replicate some of features. Therefore, translational aspects should be carefully interpreted with respect to these issues.

## Figures and Tables

**Table 1:** Biomaterials loaded with growth factors and cytokines for cardiac tissue regeneration.

Biomaterial	Growth Factors/Cytokine	Effect	References
Heparan sulfate proteoglycans	bFGF	Extended bioavailability of the growth factor by protecting it from degradation, and improved angiogenesis and cardiac function	[53]
Glutathione-modified collagen hydrogel	bFGF fused with glutathione-S-transferase and MMP-2/9 cleavable peptide TIMP	Decreased collagen deposition, increased vascularization, and improved heart function	[54]
Hydroxyethyl methacrylate hyaluronic acid hydrogel	Neuregulin-1β	Improved ventricular function and structure	[38]
Fibrin gel/heparine coacervate	VEGF and PDGF	Improved angiogenesis and cardiac function, and reduced scar formation and inflammation	[57]
Citrate-based polyester hydrogel	Mydgf	Reduced cell apoptosis and scar formation, and improved angiogenesis and cardiac function	[65]
Laponite/gelatin hydrogel	ADSC secretome	Improved angiogenesis, ejection fraction, and cardiac output, and reduced fibrosis	[66]
Poly(lactic-co-glycolic acid)–poly(ethylene glycol) nanoparticles	Liraglutide	Improved heart function, attenuated adverse cardiac remodeling, stimulated angiogenesis, and suppressed cardiomyocyte apoptosis	[70]
A sulfonated hydrogel and poly(ethylene glycol)-blockpoly(serinol hexamethylene urea)-block-poly(ethylene glycol) micelle nanoparticles	VEGF, IL-10, and PDGF	Improved angiogenesis and demonstrated potential amelioration of inflammation to optimize cardiac repair post-MI	[12]
Hyaluronic acid-based hydrogel	HGFdf and ESA	Decreased infarct size, and improved angiogenesis and heart function	[71]
Calcium-alginate microsphere patch	VEGF	Improved tissue regeneration and cardiac function, and increased capillary density	[75]
Human cardiomyocyte patch with polylactic-co-glycolic acid nanoparticles	FGF1 and CHIR99021	Reduced infarction size and improved angiogenesis and cardiac function. The combination of factors reduced apoptosis and increased proliferation of transplanted cardiomyocytes	[76]

**Table 2:** Biomaterials loaded with stem cells for cardiac tissue regeneration.

Biomaterial	Stem Cells	Effect	References
Mussel adhesive protein/HA coacervate	MSCs	Increased MSCs survival and retention	[92]
Collagen-based hydrogel	ADSCs	Improved engraftment of stem cells	[93]
Microcapsules made of agarose and ECM components	MSCs	Increased MSCs survival	[94]
Arginylglycylaspartic acid (RGD) modified HA hydrogel	MSCs	Increased MSCs survival	[95]
Erythropoietin linked hydrogel	iPSCs	Improved the post-MI heart recovery	[96]
Synthetic SAP and angiopoetin-1-derived pro-survival peptide QHREDGS	MSCs	Increased cells survival and cardiac function	[97]
Collagen–dendrimer	CPCs	Increased long-term survival	[99]
Silica-coated SOMag5 magnetic nanoparticles	Embryonic cardiomyocytes, embryonic stem cell-derived cardiomyocytes and BMSCs	Improved cell engraftment	[100]
Graphene oxide/alginate microgel	MSCs	Improved MI recovery	[101]
Poly(ε-caprolactone)/gelatin patch	MSCs	Increased angiogenesis, lymphangiogenesis, cardiomyogenesis, and paracrine factors released by stem cells and reduced scar size	[103]
Chitosan and silk fibroin microfibrous cardiac patch	MSCs	Increased MSC survival	[104]
Hyaluronic acid/gelatin cardiac patch	CPCs	Increased long-term CPCs survival and differentiation	[105]
Vascularized fibrin hydrogel patch	CSCs	Increased cell survival	[106]
Gold nanoparticles coated with a combination of ECM and silk proteins	MSCs	Increased cell survival and retention and decreased infarct size	[107]
ECM scaffold with the usage of methacrylated gelatin	iPSC-derived cardiomyocytes, smooth muscle cells and endothelial cells	Reduced infarct size and improved cell proliferation, cardiac function, and angiogenesis	[108]
Poly(vinyl alcohol) microneedle patch	CSCs	Improved angiogenesis, reduced fibrosis, and repaired left ventricular wall	[109]
Polycaprolactone patch	MSCs and iPSC-derived cardiomyocytes	Improved MI recovery and angiogenesis	[110]
Microchanneled poly(ethylene glycol) dimethacrylate hydrogel patch	MSCs	Improved cardiac function	[112]
Agarose hydrogel microcapsules supplemented with fibronectin and fibrinogen	CSCs	Improved production of pro-angiogenic/cardioprotective cytokines, angiogenesis, and angiogenic cells recruitment after direct intramyocardial injection	[113]
Iron nanoparticles	MSCs	Reduced the infarct size, prevented fibrosis, decreased apoptosis of myocardial cells, increased angiogenesis, and improved cardiac function	[115]
Statin-conjugated poly(lactic-co-glycolic acid) nanoparticles	ADSCs	Increased the ejection fraction and several other parameters which reflect the left ventricular function. Inhibited local inflammation, promoted recruitment of circulating stem cells, and stimulated their differentiation to cardiomyocytes and angiogenesis	[121]
Tetraaniline-polyethylene glycol diacrylate and thiolated hyaluronic acid conductive hydrogel	ADSCs	Improved neovascularization, regeneration of the damaged myocardium, and post-infarction cardiac function	[17]
Naphthalene hydrogel	MSCs	Increased cells survival, and stimulated the synthesis of angiogenic factors VEGF and SDF-1α	[122]
Poly(lactic-co-glycolic acid) microparticles	ADSCs	Improved cells survival, and induced the shift of macrophage found in the infarcted myocardium from pro-inflammatory M1 to regenerative M2 phenotype	[123]
Poly(l-lactic acid) mat	CSCs	Improved angiogenic and cardiomyogenic effects	[124]
